# 18S rRNA V9 metabarcoding for diet characterization: a critical evaluation with two sympatric zooplanktivorous fish species

**DOI:** 10.1002/ece3.1986

**Published:** 2016-02-19

**Authors:** Aitor Albaina, Mikel Aguirre, David Abad, María Santos, Andone Estonba

**Affiliations:** ^1^ Laboratory of GeneticsDepartment of Genetics, Physical Anthropology & Animal PhysiologyUniversity of the Basque Country (UPV/EHU)Leioa48940Spain; ^2^ Marine Research DivisionAZTI TecnaliaHerrera Kaia Portualdea z/gP.O. Box 20110PasaiaGipuzkoaSpain

**Keywords:** 18S rRNA V9, diet analysis, metabarcoding, MiSeq paired‐end technology, plankton, *Sardina pilchardus*, *Sprattus sprattus*

## Abstract

The potential of the 18S rRNA V9 metabarcoding approach for diet assessment was explored using MiSeq paired‐end (PE; 2 × 150 bp) technology. To critically evaluate the method′s performance with degraded/digested DNA, the diets of two zooplanktivorous fish species from the Bay of Biscay, European sardine (*Sardina pilchardus*) and European sprat (*Sprattus sprattus*), were analysed. The taxonomic resolution and quantitative potential of the 18S V9 metabarcoding was first assessed both *in silico* and with mock and field plankton samples. Our method was capable of discriminating species within the reference database in a reliable way providing there was at least one variable position in the 18S V9 region. Furthermore, it successfully discriminated diet between both fish species, including habitat and diel differences among sardines, overcoming some of the limitations of traditional visual‐based diet analysis methods. The high sensitivity and semi‐quantitative nature of the 18S V9 metabarcoding approach was supported by both visual microscopy and qPCR‐based results. This molecular approach provides an alternative cost and time effective tool for food‐web analysis.

## Introduction

Improvements in our understanding of marine food‐webs are required for Ecosystem‐Based Fisheries Management (EBFM) (i.e. Rice 2011; Gallego et al. 2012). Multispecies food‐web modeling is increasingly being used in fisheries management and such models are often heavily dependent upon accurate depictions of diet (Plagányi et al. 2014). This involves identifying the dietary breadth and, ideally, quantifying the relative contributions of prey taxa. Developing accurate techniques for determining dietary components is critical for this endeavour but these techniques must also be cost and time effective (Pompanon et al. 2012). With the advent of high throughput sequencing (HTS) technologies, the metabarcoding approach, where one or few DNA regions (barcodes) are sequenced for every organism within a sample, has the potential to provide a significant step‐change in the analysis of food‐webs. Metabarcoding can resolve previously unknown trophic relationships involving organisms which were traditionally difficult or impossible to identify using visual methods (Pompanon et al. 2012; Symondson and Harwood 2014). In addition the technique is highly sensitive and is capable of detecting traces of DNA in community samples, such as plankton (e.g. Eiler et al. 2013; Lindeque et al. 2013; Pochon et al. 2013; Zhan et al. 2013, 2014; Brown et al. 2015; Hirai et al. 2015).

Although molecular methods have often only been used to give presence/absence information, the metabarcoding can be applied semiquantitatively to estimate relative abundances within a sample (e.g. Amend et al. 2010; Murray et al. 2011). The 18S rRNA V9 barcode (18S V9 hereinafter) is potentially ideal for diet assessment purposes, typically involving degraded DNA and a relatively broad spectrum of prey, because it has (1) a broad amplification range (e.g. Amaral‐Zettler et al. 2009), (2) a multicopy nature, (3) a relatively short amplicon size (~160–170 bp), and (4) an extensive representation in public databases (e.g. King et al. 2008; Pompanon et al. 2012). Due to these favorable characteristics, 18S V9 metabarcoding has been already used in diet assessment (O'Rorke et al. 2012, 2013, 2015; Jarman et al. 2013).

However, none of these previous efforts applied the Illumina′s MiSeq paired‐end sequencing technology (MiSeq PE) that has significantly lowered the costs per base. Furthermore, MiSeq PE approach allows performing bidirectional sequencing (hereinafter: the complete overlap of the amplicon from both forward and reverse sequencing senses) of 150–250 bp amplicons. Yet, a critical evaluation of the performance and quantitative nature of 18S V9 metabarcoding technique is lacking, for example, it has not been compared to established methods such as microscopy or qPCR.

Here, we evaluate the potential of the MiSeq PE based 18S V9 metabarcoding technique to analyze digested/degraded DNA. To address this, we firstly evaluated its performance against taxonomically characterized mock and field plankton samples. This was a necessary step for further application to analyzing the stomach contents of two species of zooplanktivorous (European sardine, *Sardina pilchardus* and European sprat, *Sprattus sprattus*) in the Bay of Biscay. These two species, along with European anchovy (*Engraulis encrasicolus*), represent the bulk of small pelagic fish inhabiting the Bay of Biscay (ICES 2010). Both species are planktivorous, with medium‐sized copepods as their main prey, but including a broad range of alternate prey (Möllmann et al. 2004; Garrido et al. 2008; Raab et al. 2012; Costalago et al. 2015). While both species inhabit the coastal waters of the Bay of Biscay, sardine are found out to the shelf‐break up to 200 nautical miles from the coast (ICES 2010). The presence of contrasting plankton communities at the different biomes occupied by sardine (Albaina and Irigoien 2004; Zarauz et al. 2008) allowed us to test the capacity of the 18S rRNA V9 metabarcoding approach to detect spatially related shifts in diet composition.

## Materials and Methods

### Field samples

**Figure 1:**
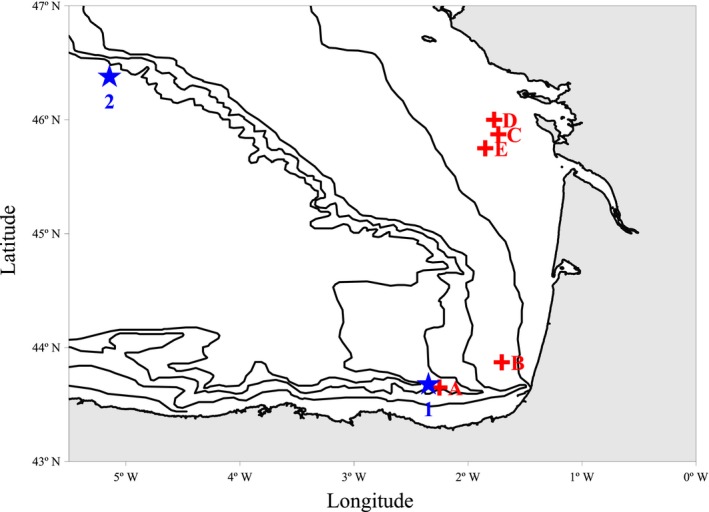
MIK and fish haul locations. Spatial locations of the field plankton samples (MIK‐1 and MIK‐2; stars) and fish hauls (A–E; crosses). Isobaths of 100, 200 1000 and 2000 m are shown. MIK‐1 and MIK‐2 were collected the 8th of May 2010 and the 22th of May 2011 (both between 3:00 and 4:00 AM; local time) respectively.

Two zooplankton samples (MIK‐1 and 2; Fig. 1) were collected in the Bay of Biscay with an oblique Methot Isaac Kidd (MIK) net tow. The net had 1 mm mesh and a mouth area of 1 m^2^ (more details in Albaina et al. 2015). Samples were preserved immediately after collection in 100% ethanol. Two aliquots of the same volume (obtained using a Motoda plankton splitter) were processed independently for microscopy and metabarcoding. Visual analysis was performed using a stereoscopic microscope and identification was made to species or genus level where possible. As reliable biomass estimation was not available for every taxon (e.g. gelatinous remains), the correlation between relative abundances retrieved by microscopy and metabarcoding was limited to the 13 taxa sorted for the mock samples (next section). Microscopy counts were transformed to biomass (μg C dry weight) using available length‐biomass regressions and assuming a 40% of carbon content in total dry weight (Bamstedt 1986). The formulas of Gaudy and Boucher (1983), Lindley et al. (1999) and Lavaniegos and Ohman (2007) were used for biomass calculations of copepods, *Meganyctiphanes norvegica* and *Tomopteris* spp. respectively.

### Mock samples

Four mock samples (MOCK‐A, B, C and D) were constructed using the remaining aliquots of the MIK samples. Known quantities of previously visually identified (and measured) organisms from 13 different species were mixed in different relative proportions (Table 1), in order to assess the capacity of metabarcoding to estimate relative species abundances. Moreover, the over‐representation of one taxon in MOCK‐D (89% of counts and 91% of biomass as *Meganyctiphanes norvegica*) allowed testing the sensitivity of the metabarcoding approach to detect the other taxa at low abundance (e.g. Quigley et al. 2014).

**Table 1:** Mock samples′ composition. Four mock samples were constructed including 884 individuals from 13 different species (MOCK‐A, B, C, and D). Individuals' average size (total length; TL) and standard deviation (SD) is shown. Eight species (shaded) shared one 18S V9 sequence and thus represented a sole OTU (“Para‐Und‐Euch group”); the range of TL and SD values for the four mock samples is shown for this specific OTU

		MOCK‐A	MOCK‐B	MOCK‐C	MOCK‐D	TL	SD
*Meganyctiphanes norvegica*	Euphausiid	101	33	1	100	8.7	2.0
Para‐Und‐Euch group	Copepod	90	270	8	8	4.7–5.1	1.3–1.5
* Undeuchaeta major*		13	39	1	1	5.5	0.2
* Undeuchaeta plumosa*		3	9	1	1	4.5	0.2
* Euchirella rostrata*		20	60	1	1	3.7	0.1
* Euchirella curticauda*		2	6	1	1	4.6	0.3
* Paraeuchaeta gracilis*		22	66	1	1	7.3	0.5
* Paraeuchaeta tonsa/pseudotonsa*		12	36	1	1	5.4	0.3
* Euchaeta hebes*		15	45	1	1	3.3	0.2
* Euchaeta acuta*		3	9	1	1	3.7	0.2
*Pleuromamma robusta*	Copepod	23	69	1	1	4.1	0.3
*Candacia armata*	Copepod	10	30	1	1	2.9	0.2
*Calanus helgolandicus*	Copepod	7	21	1	1	3.0	0.2
*Tomopteris* spp.	Polychaeta	25	80	1	1	7.6	1.1

### Sardine and sprat diet assessment

The diet of 64 adult sardine and 20 adult sprat were characterized using the 18S V9 metabarcoding approach. Fish were collected by pelagic trawling in five different hauls distributed across the shelf of the Bay of Biscay in May 2010 (Fig. 1 and Table S1; more details in Albaina et al. 2015). Haul distribution covered the two species′ potential habitat in the Bay of Biscay (ICES 2010). Sardine were found at the shelf‐break (haul A; 6th May 2010), external (haul B, 9th May) and internal continental shelf (haul C and D, respectively, 15 and 16th May) while sprat were collected from the latter location (haul E, 18th May). Fishing was performed during daylight except for haul D which took place at midnight. Animals were kept frozen (−20°C) until dissection and stomach contents preserved in 100% ethanol until DNA extraction. Dissecting tools were flame sterilized after each dissection and 12 extraction blank controls (negative control, where no tissue is added to the extraction buffer prior to DNA extraction protocol) were included to detect cross‐contamination.

## 18S V9 in silico test for taxonomic resolution

The primers developed in the framework of the Earth Microbiome Project (EMP) for 18S rRNA amplification were used (Illumina_Euk_1391f and Illumina_EukBr; http://www.earthmicrobiome.org/emp-standard-protocols/18s/). These primers target the V9 region of the 18S rRNA gene. Twenty‐nine individuals from 13 zooplankton and four clupeid fish species from the Bay of Biscay and surrounding waters were sequenced for the 18S V9 region (Table S2; GenBank accession numbers KP768123‐KP768151). DNA was extracted following the salt extraction method (Aljanabi and Martinez 1997). The 18S V9 region was amplified in a 25 μL reaction containing 2.5 μL of each primer (10 μmol/L), 2.5 μL dNTP (0.2 mmol/L of each dNTP), 2.5 units of GoTaq polymerase (5 u/μL; Promega), 2.5 μL MgCl_2_ (25 mmol/L), 0.5 μL BSA (10 mg/mL; New England BioLabs), 5 μL 5× Buffer, 8 μL Milli‐Q water (Merck, Darmstadt, Germany), and 1 μL of DNA extract. Reactions were amplified through 35 cycles (30 s at 95°C, 45 s at 57°C and 45 s at 72°C) followed by a final extension step of 7 min at 72°C. The purified PCR products were sequenced in both directions on an ABI 31309 capillary electrophoresis Analyzer with ABI BigDye Terminator version 3.1 chemistry (Applied Biosystems, Foster City, CA).

In order to assess the taxonomic resolution of the 18S V9 region, these sequences were combined with forty‐eight individual 18S V9 sequences including 12 copepod and eight euphausiid species inhabiting North East Atlantic (NEA) waters (respectively, Laakmann et al. 2013 and GenBank popset 117414780; Table S2). The sequences were aligned using MUSCLE (Edgar 2004) implemented in MEGA5 (Tamura et al. 2011). Based on the resulting multiple sequence alignment (MSA), a maximum likelihood (ML) tree was constructed with MEGA5 following the guidelines of Hall (2013) in order to depict intra‐ and interspecies variability.

These sequences, along with another seven individual sequences from taxa commonly found in the Bay of Biscay plankton community and within zooplanktivorous fish diets, were added to the SILVA database (v111; clustered at 99% identity) to construct a custom database. In total 42 species were included to complete the reference database (Table S2) used for the taxonomic assignment of the metabarcoding data. . Using our custom database the assignment success increased from 65% (SILVA v111 alone) to 76.5% using a 99% identity threshold. Finally, in order to further describe the capacity of 18S V9 barcode to resolve species‐level assignments and to avoid redundancy in the database, the cases of synonymy between the 42 sequences that we added in our custom database (presented in Table S2) and the sequences of the SILVA database were explored. For this exploration, the BLASTN algorithm was used with default parameters and only the hits featuring 100% identity over a minimum length of 64 nucleotides were considered.

## 18S V9 metabarcoding

The total DNA content of 84 fish stomachs was extracted following Albaina et al. (2015; see for protocol details). DNA was extracted from the four mock and the two field plankton samples by centrifuging the samples (50 mL Falcon tubes) at 3488 × g for 30 min to pellet the organisms. Absolute ethanol was removed before beginning the DNA extraction protocol. The quantity and quality of extracted DNA was assessed using either an ND‐1000 spectrophotometer (NanoDrop; stomach contents) or Qubit fluorimeter (Life technologies; plankton samples). To avoid cross‐contamination, disposable filter tips were used in every step, dissection tools were flame sterilized between individuals and lab surfaces were decontaminated with DNA‐ExitusPlus (Applichem, Darmstadt, Germany) after each DNA extraction session. Extracted DNA was shipped on dry ice to Argonne National Laboratory (IL, USA) where the 18S V9 region was sequenced according to the EMP protocol, without including blocking primers (see Caporaso et al. 2012 for further details). A total of 146 individually tagged libraries were constructed using distinct 12 bp tags added to reverse primers. One microliter of extracted DNA (~10–100 ng) was added to each PCR reaction and three PCR replicates were pooled per library. Following quantification by Picogreen (Invitrogen), amplicon libraries were pooled in equimolar concentrations for sequencing. In order to test reproducibility and to evaluate the effect of random sampling in the quantitative value of metabarcoding (Zhou et al. 2011), technical replicates were included. A total of five different libraries were constructed for each mock and field DNA sample (20 and 10 libraries respectively). Moreover, a second library was also independently sequenced for 32 (out of 84) of the fish stomach extracts. Including these, a total of 162 amplicon libraries were sequenced distributed in two runs of Illumina MiSeq (PE reads; 2 × 150 bp) including two no template controls per run and the 12 extraction blank controls. A 30–50% PhiX DNA was added to each run as to improve data quality.

### Bioinformatics pipeline

Raw sequences were quality trimmed using Sickle v1.33 (Joshi and Fass 2011) with default parameters (including Phred score ≥20). Next, paired‐end reads were aligned and merged with PEAR software v 0.9.6 (Zhang et al. 2014) using a cut‐off of 0.01 (*P*‐value) for the observed expected alignment score allowing one mismatch in the aligned region and prioritizing the highest Phred score base. It has to be noted that the whole variable region of the 18S V9 was totally overlapped (bidirectional sequenced) by the 2 × 150 bp sequencing strategy. By querying the full alignment with this stringent premises, we expected to retrieve full high‐quality 18S V9 sequences. Sequences with incorrect barcodes were removed by fastq‐barcode.pl (Smith 2012) prior to entering QIIME software v 1.8 (Caporaso et al. 2012) for further analyses. Chimeric sequences were removed using the UCHIME v4.2 method (Edgar et al. 2011) using both reference and de‐novo strategies. Then, sequences were assigned taxonomy with UCLUST v1.2.22q (Edgar 2010) using both the closed and open reference methods with a 97, 99, and 100% identity threshold against our custom reference database. While the identity threshold corresponds to the minimum required to assign taxonomy, UCLUST prioritizes the match with the highest identity if >1 fulfils it. Thus, providing the target species is in the barcode database, miss‐assignments would be only possible in the case of an amplicon synonymy (100% identity between two species) or a sequencing error. Following taxonomic assignment, singletons were removed and the unassigned reads (in the closed reference method) were collapsed into a new category. While the closed reference method only considers OTUs within the reference database, the open reference method clusters by similarity also those sequences with no database correspondence (i.e. Rideout et al. 2014). The Kruskal**–**Wallis (KW) test was applied to evaluate the technical replicates similarity. The Pearson correlation coefficient was used for the comparison of microscopy (counts and biomass) results against metabarcoding ones. Regarding mock and field samples, we distinguished between the “within taxon” quantification (the capacity of retrieving relative abundance for a certain taxon within different sample compositions) and the “among taxa” one (the capacity of obtaining relative abundances for every taxon within a certain sample).

Finally, predator reads were removed from fish stomach samples and only OTUs showing abundances >0.5% in at least one sample were considered for the DCA (detrended correspondence analysis) using version 4.5 of CANOCO (ter Braak and Smilauer 2002) applied to square‐root transformed relative abundances.

### Comparison against species‐specific qPCR assay

The sensitivity of metabarcoding to detect digested DNA was compared against a qPCR approach designed to identify European anchovy (*Engraulis encrasicolus*) DNA within predator stomach contents. TaqMans assays targeting an 87 bp region of the mitochondrial DNA cytochrome‐b (mtDNA cytB) and capable of detecting as few as 0.005 ng of *E. encrasicolus* DNA were performed (Albaina et al. 2015). Only those stomachs giving a positive signal for the qPCR assay (*n* = 60) were considered for comparison against metabarcoding data.

As a sole base change in the 18S V9 could imply a species miss‐assignment between anchovy and the predator species (one nucleotide variation between anchovy and sprat, two between anchovy and sardine and three when comparing both predator species; Fig. S1), a custom script (Aguirre 2015) was created to validate assignments using a character‐based discrimination approach (using diagnostic SNPs as in Jaén‐Molina et al. 2015). The script was applied to validate clupeid assignments (herring, sardine, sprat, and anchovy) in our metabarcoding data. By querying only the three diagnostic 18S V9 nucleotides for these four species (Fig. S1) we expect a negligible effect of the machine sequencing error on the clupeid species assignment in the character‐based approach. We first retrieved every read assigned as clupeid by the bioinformatics pipeline (UCLUST and closed reference method). Then, applying the custom script these reads were first aligned and then the nucleotides at the three diagnostic positions were used to perform the species identification. The qPCR – metabarcoding correlation (Pearson correlation coefficient) was calculated with both sets of data (clupeid species repartition with or without running the character‐based method script).

Finally, it has to be noted that we are comparing a region of 87 bp in the multicopy mtDNA cytB gene against a 174 bp (for clupeid fish including primers) region in the multicopy nuclear 18S rRNA gene. Assuming both sets of primers amplify the target DNA with similar efficiency, detectability under digestive process is affected by both the distinct size (a shorter amplicon is detected for longer digestion times, Deagle et al. 2006), but also by the distinct number of copies per cell which is associated with both mitochondrial and ribosomal DNA (Gibbons et al. 2014). However, given the scarcity of studies comparing qPCR and metabarcoding performances with digested DNA samples (Murray et al. 2011), this analysis gave insights into (1) the sensitivity of the 18S V9 metabarcoding approach and, (2) its capacity to serve as a proxy for prey abundance. We compared the percentage of *E. encrasicolus* DNA in relation to total extracted DNA (as in Albaina et al. 2015) against the percentage of 18S V9 reads for this species.

### Bidirectional (2 × 150) versus single‐direction (1 × 150 bp) 18S V9 amplicon sequencing

Bidirectional sequencing is capable of producing longer and higher quality reads by applying a stringent PE reads merging step in order to correcting nucleotide ambiguities (keeping the best quality nucleotide in any of both overlapping reads) (e.g. Eren et al. 2013). This is critical when aiming high taxonomical resolution and, especially, when dealing with partially degraded DNA such as with diet remains. We evaluated the effect of bidirectional sequencing in the assignment success by focusing on the subset of reads assigned to clupeid taxa (using the closed reference method and a 99% identity threshold for taxonomic assignment). Apart from the large number of reads belonging to both predator species (sardines and sprats) and the already detected presence of European anchovy DNA remains within the studied stomach contents (Albaina et al. 2015), the low number of variable positions between these closely related species (see above) makes them a perfect subject for the evaluation of assignment performance.

In order to generate OTUs that would be obtained based on a single‐end sequencing run, only the reads that correspond to the 5′‐end of the amplified fragment, and thus entailing the forward amplification primer were retained. These sequences were then treated as for the sequences resulting from the assembly of the paired reads obtained based on the PE sequencing run, that is using both UCLUST and the above described character‐based assignment methods. The same bioinformatics pipeline of the PE reads was applied to single‐end reads except for the merging step. We then compared the degree of discrepancy between both assignment methods in both the bidirectional (PE reads) and single‐direction sequencing approaches. In theory, the assignment discrepancy against the character‐based approach for a certain species should be lower in the bidirectional sequencing than in the single‐direction one. Apart from this, the total number of assigned reads should be higher in the former due to a longer amplicon allowing a higher taxonomic resolution.

## Results

### 18S V9 in silico test for taxonomic resolution

Seventy‐seven 18S V9 sequences from thirty‐five taxa (twenty‐three copepods, eight euphausiids, four clupeid fish, and one annelid species) were included in the analysis (Table S2; Fig. S2). Amplicon length (without primers) ranged from 120–134 bp. In relation to intraspecies diversity, no nucleotide variation was observed (up to five individuals per species). While the 18S V9 was able to discriminate congeneric copepod species of the *Acartia* and *Pseudocalanus* genus, a 100% identity corresponded to the two *Centropages* species tested and, more interestingly, to eight copepod species including representatives from two different families, Aetideidae and Euchaetidae (*Paraeuchaeta gracilis*,* Paraeuchaeta tonsa/pseudotonsa*,* Undeuchaeta plumosa*,* U. major, Euchaeta hebes*,* Euchaeta acuta*,* Euchirella rostrata,* and *Euchirella curticauda*; from now onwards “Para‐Und‐Euch group”). Apart from this, five different euphausiid species had identical sequences (*Meganyctiphanes norvegica*,* Nyctiphanes simplex*,* Tessarabrachion oculatum*,* Nematobrachion flexipes,* and *Stylocheiron carinatum*) whilst the remaining euphausiid species were discriminated at one or two positions. For the clupeid fish species, three variable positions allowed the distinction of the four species involved (Fig. S1).

When aligning the sequences added to the custom database (Table S2) against SILVA database, a 100% identity in the 18S V9 region was detected for *Clupea harengus* (one teleost hit), *Candacia armata* (three hits; three congeneric species), *Calanus helgolandicus* (four Calanidae hits, including one congeneric species) and *Pleuromamma robusta* (two hits, two congeneric species) (Table S3).

### Taxonomic assignment identity threshold

**Figure 2:**
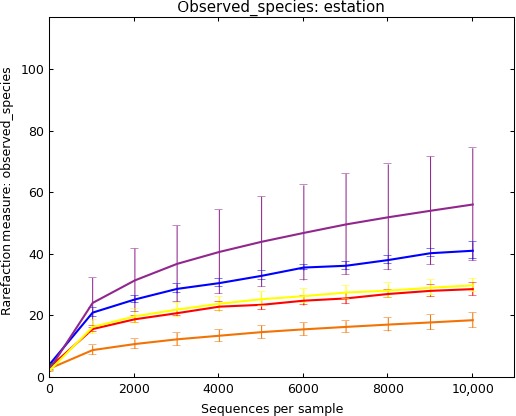
Rarefaction curves. Alpha rarefaction plot generated with QIIME at the 99% identity threshold. Observed species (OTUs; left axis) plotted against sequencing depth (bottom axis; limited to the first 10,000 reads). The different colored curves represent sardine and sprat stomach contents (purple and yellow, respectively), mock (orange), MIK‐1 (red) and MIK‐2 (blue) samples.

Eleven percent of the single‐end reads were discarded in the PE merging step. Considering all sequenced samples together (mock and field plankton samples along with fish stomach samples), a total of 13,090,614 merged reads passed quality filters, an average of 88,000 reads per sample when excluding controls. Regarding controls, this reduced to a mere 800 and 20 reads for, respectively, extraction blank controls (50% of reads corresponding in average to unassigned and Kingdom Fungi sequences) and no template ones (90% unassigned and Kingdom Fungi sequences Only 0.03% of reads were removed due to their putative chimeric nature, and 193 OTUs (0.001% of the reads) were also removed as they corresponded to singletons. The majority of the remaining reads were assigned taxonomy by the closed reference method (86.7%), which at threshold of 97% sequence identity resulted in a total of 809 OTUs across the mock, field and fish stomach samples. At thresholds of 99 and 100%, the number of delineated OTUs reduced to 528 (76.5% of reads) and to 357 (60.7% reads) respectively. However, given the taxonomic resolution of the 18S V9 region, a 99% identity threshold was considered as optimum due to its stringency (but below the machine error rate, <1%, Quail et al. 2012). A sufficient sequencing depth was evident for each sample type, as illustrated by the rarefaction curves (Fig. 2). As expected, a lower sequencing effort was needed to capture the sequence diversity of the mock samples as compared to the sardine diet samples. Finally, the open reference method yielded a total of 1356, 1634, and 2291 OTUs for 97, 99, and 100% identity thresholds respectively. However, due to the lack of a reference sequence in public database and thus in the absence of comparable taxonomic and ecological information for a significant proportion of those OTUs (28, 65, and 84% for the 97, 99, and 100% identity thresholds, respectively), we focused our further analyses only on the OTU dataset generated by the closed reference clustering (and the 99% identity threshold ones as reasoned above).

### Mock samples metabarcoding

**Figure 3:**
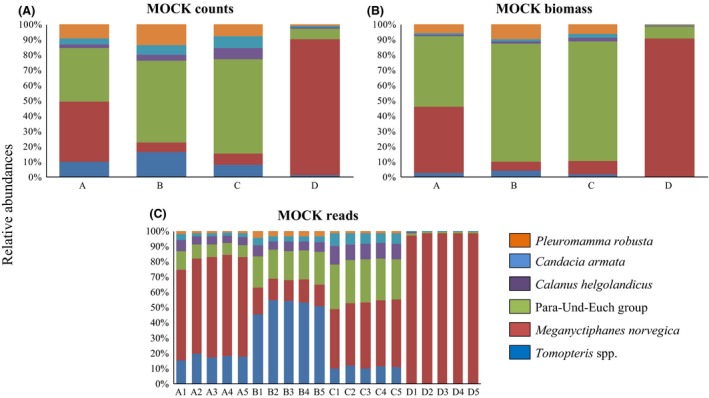
Mock samples. Relative abundance of (A) microscopy counts, (B) estimated biomass (C dry weight) and, (C) 18S V9 reads, for the six OTUs within mock samples. Five technical replicates were sequenced (1–5; bottom graph). No bias in OTUs distribution was reported for the technical replicates (Kruskal**–**Wallis test). Legend superimposed.

Individuals of similar size were sorted for the construction of the mock samples (Table 1). Hence, the species proportions expressed in terms of biomass and specimens counts could be correlated (Pearson correlation *r* = 0.98–1; *P* < 0.01). Due to the 100% identity among the sequences of the different species composing the “Para‐Und‐Euch” group, a single OTU corresponded to those eight species in the metabarcoding analysis (Fig. 3C). A total of 134 OTUs were reported from the mock samples. Only six OTUs corresponded to taxa related to the species that were sorted from the MIK samples and sequenced. These six OTUs gathered 89.2% of the reads, whilst 10.3% of the reads were not assigned. The remaining 0.5% of reads were distributed among 128 OTUs. The six OTUs corresponding to the sorted taxa were recorded by the metabarcoding approach in every sequenced library except for that corresponding to *Pleuromamma robusta*, which was absent in three of the five technical replicates of MOCK‐D. These three false negatives corresponded to replicate samples characterized by a relatively low sequencing depth (an average of 27,500 sequences per sample against ~52,500 reads in the remaining two replicates). Comparing relative abundances among taxa (relative abundances of every taxon within each particular sample), a significant correlation between the metabarcoding (18S V9 sequence counts) and microscopy approach was only recorded for MOCK‐D sample (both against microscopy counts and biomass; *r* = 0.99 and *P* < 0.01). Within taxon (each particular taxon′s relative abundances at the four sample settings), significant correlations were reported for every taxon (18S V9 counts both against microscopy counts and biomass; *r* = 0.94–0.98, *P* < 0.05) except for *Tomopteris* spp. where this only applied to the latter (*r* = 0.95).

### Field samples metabarcoding

A total of 133 OTUs were detected in field samples (93 and 102 in MIK‐1 and MIK‐2, respectively) whilst only 43 taxa were identified visually (36 and 33 respectively). Table 2 shows the taxa with ≥1% abundances in microscopy counts and/or metabarcoding analysis. Not considering unassigned reads (23 and 44% in MIK‐1 and MIK‐2, respectively), the most abundant OTUs corresponded well with those taxa identified visually. Regarding MIK 1, *M. norvegica* representing 30% of counts attained a 50% of 18S V9 reads. Besides this, *Calanus helgolandicus*, the “Para‐Und‐Euch” group and *Tomopteris* spp., were abundant in both approaches. Apart from this, *Candacia armata* with 2.1% of the counts showed 0.14% of the 18S V9 reads. On the other hand, *M. norvegica* comprised 4.8% of the visual counts and 18.4% of the 18S V9 reads in the MIK‐2 sample where, another euphausiid taxon not represented in the reference database, *Nematoscelis megalops*, attained 9.1% of the counts. Apart from this, *Calanus helgolandicus*, the “Para‐Und‐Euch” group and *Pleuromamma robusta* were abundant in both the metabarcoding and microscopy datasets.

**Table 2:** Field samples; comparison between metabarcoding and microscopy results. OTUs comprising at least 1% of total abundance in either metabarcoding (% reads; left columns) or microscopy (% counts, right one) analysis are shown ranked in abundance for both MIK‐1 and MIK‐2 (top and bottom rows respectively). Average read proportion and standard deviation (SD) for the five technical replicates are shown for the metabarcoding approach. No bias in OTUs distribution was reported for the technical replicates (Kruskal**–**Wallis test)

MIK‐1 (metabarcoding 18S V9)	Reads (%)	SD	MIK‐1 (microscopy)	Counts (%)
*Meganyctiphanes norvegica*	49.87	2.88	*Meganyctiphanes norvegica*	30.29
Unassigned	22.61	1.47	Gelatinous organisms	27.18
Hydrozoa; uncultured eukaryote	8.07	0.41	Para‐Und‐Euch group	20.00
*Tomopteris* spp.	6.31	0.58	*Tomopteris* spp.	4.27
Para‐Und‐Euch group	5.83	0.61	Others	3.11
*Salpa fusiformis*	3.03	0.44	*Calanus helgolandicus*	2.33
*Calanus helgolandicus*	1.83	0.22	*Candacia armata*	2.14
			Myctophidae	1.55

MIK‐2 (metabarcoding 18S V9)	Reads (%)	SD	MIK‐2 (microscopy)	Counts (%)
Unassigned	43.90	0.51	Gelatinous organisms	53.87
*Meganyctiphanes norvegica*	18.44	1.30	Para‐Und‐Euch group	15.74
Hydrozoa; uncultured_eukaryote	18.36	0.67	*Nematoscelis megalops*	9.08
Syndiniales Group I; uncultured eukaryote	4.79	0.29	*Pleuromamma robusta*	8.11
*Calanus helgolandicus*	3.80	0.33	*Meganyctiphanes norvegica*	4.84
Para‐Und‐Euch group	3.77	0.28	Fish eggs	1.21
*Pleuromamma robusta*	2.68	0.20	*Calanus helgolandicus*	1.21
*Salpa fusiformis*	1.37	0.13	Myctophidae	1.21

The taxa selected for biomass estimation represented 60 and 31% of the specimen count in the MIK‐1 and MIK‐2 samples, respectively. Their counts and biomass proportions were significantly correlated in MIK‐1 (*r* = 0.99, *P* < 0.01) but not in MIK‐2 (*r* = 0.78, *P* = 0.07). This could correspond to a larger proportion of *M. norvegica* in biomass when compared with counts in the MIK‐2 sample (Fig. S3). Interestingly, the average size of *M. norvegica* (11.7 mm in length) was higher in the MIK‐2 samples as compared to the MIK‐1 samples (9.5 mm). When testing the proportions among them in each MIK sample, both microscopy counts and biomass proportions were significantly correlated with 18S V9 sequences one in MIK‐1 (respectively, *r* = 0.85–0.87, *P* < 0.05 and 0.83–0.86, *P* < 0.05). However, MIK‐2 correlation coefficients were 0.10–0.15 (*P* = 0.77–0.84) and 0.68–0.71 (*P* = 0.11–0.14) when comparing 18S V9 sequences proportion against those for microscopy counts and biomasses respectively.

### Sardine and sprat diet characterization

**Figure 4:**
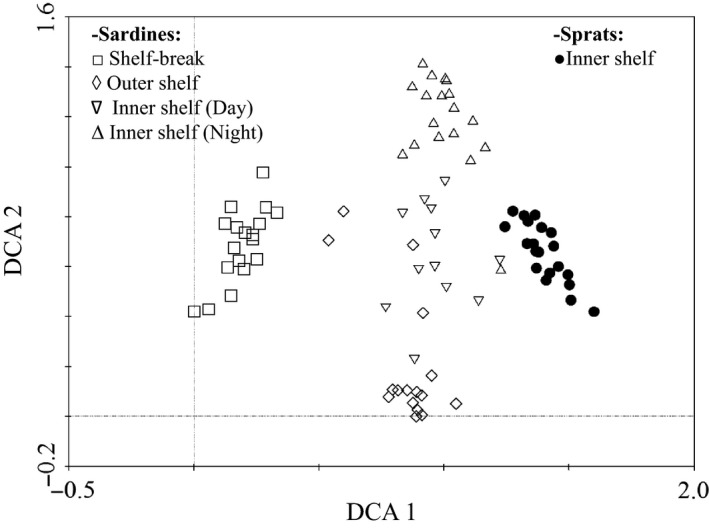
Multivariate analysis of sardine and sprat diets as determined by 18S V9 metabarcoding. Detrended correspondence analysis (DCA) generated considering OTUs comprising ≥0.5% of 18S V9 reads within any of the analyzed stomachs (a total of 37 OTUs). The five fish hauls (*n* = 84 stomachs) are represented with distinct symbols; library replicates were not included in this analysis (see Figure S5). Empty squares corresponded to shelf‐break collected sardines (haul A), rhombus did to outer shelf ones (haul B) and down‐ and up‐oriented triangles represented, respectively, haul C and D sardines (both in the inner shelf area). Finally, the sole sprat haul (inner shelf, haul E) was represented by black dots. Fishing was performed during daylight except for haul D which took place at midnight.

**Figure 5:**
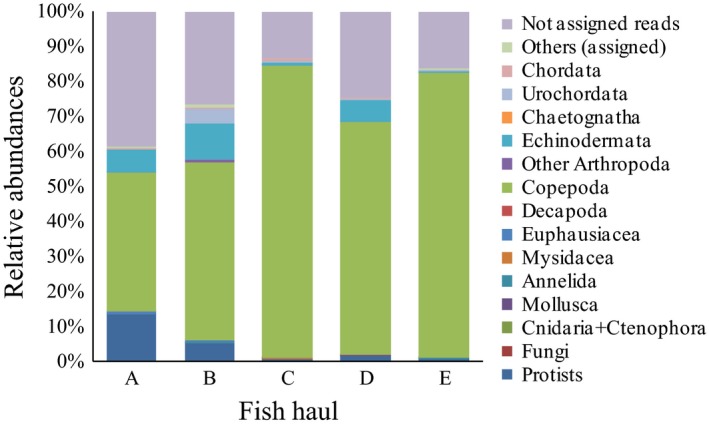
Fish diet composition. Diet composition of sardines (hauls A‐D) and sprats (haul E) as determined by 18S V9 metabarcoding. Legend superimposed.

A total of 3.4% of the assigned reads from the fish stomach samples corresponded to predator DNA (an average of 2.6 and 4.7% for sardine and sprat stomachs, respectively) and were excluded from further analyses. The detrended correspondence analysis (DCA) discriminated the diet of the two species and also the different hauls for sardine (Fig. 4). While sprat and sardines collected from the shelf break showed the most contrasting diets, a cross‐shelf gradient was also evident for sardine diet. The two sardine hauls in the inner shelf domain, corresponding to daytime and nighttime hauls were also differentiated (haul C and D respectively). Although hauls C and D showed some overlap, when performing the Mann**–**Whitney U test for both samples, more than half of the OTUs (21 of 37) presented significantly different abundance distributions. The same pattern was reported when considering the open reference method′s results (see Materials and Methods; Fig. S4). Between three to nine OTUs, from a total of 481 detected in the fish guts (474 and 155 for sardine and sprat respectively), comprised the bulk of 18S V9 reads in the five sampled hauls (Table 3). Copepods were the main prey item (Fig. 5) with two species, *Calanus helgolandicus* and *Temora longicornis,* representing between 36 to ~80% of total 18S V9 reads. The relative abundance of “unassigned reads” ranged from 13 to 35%. The remaining taxonomic groups showing ≥1% of 18S V9 reads belonged to Echinodermata, Urochordata, as well as to protists, including Archaeplastidae, Stramenopiles, Alveolates, Rhizaria, Cryptophyceae, and Haptophyta). Finally, a DCA plot of all stomach technical replicates showed that replicates were typically adjacent to each other on the plot indicating no bias in the library preparation process (Fig. S5).

**Table 3:** Sardine and sprat diet characterization by means of 18S V9 metabarcoding. Data for the 13 OTUs showing, on average, ≥1% of 18S V9 reads within any fish haul (highlighted) are shown. The total number of OTUs recorded for each haul is also shown along with the number of stomachs analyzed and fishing light conditions. NA stands for Not Available

Fish haul		Haul A	Haul B	Haul C	Haul D	Haul E
Predator		Sardina pilchardus				Sprattus sprattus
Light conditions		Day	Day	Day	Night	Day
Stomachs (n)		18	16	18	12	20
*Calanus helgolandicus*	Copepoda	35.51	16.40	78.22	45.31	32.26
*Temora longicornis*	Copepoda	0.42	34.37	5.36	17.45	47.49
Unassigned	NA	34.96	26.49	12.67	23.54	16.53
Syndiniales Group I; uncultured eukaryote	Protists	10.64	1.48	0.06	0.60	0.10
*Spatangus raschi*	Echinodermata	0.23	4.07	0.13	4.58	0.00
*Leptosynapta clarki*	Echinodermata	6.58	5.40	0.49	2.74	0.06
*Oikopleura dioica*	Urochordata	0.11	3.53	0.38	0.76	0.53
Para‐Und‐Euch group	Copepoda	2.98	0.31	0.01	0.01	0.01
*Centropages typicus*	Copepoda	2.63	0.22	0.11	1.82	0.33
Syndiniales Group I; uncultured dinoflagellate	Protists	0.11	1.13	0.07	0.17	0.00
Syndiniales Group I; uncultured marine eukaryote	Protists	0.05	1.04	0.12	0.07	0.00
Calanoid copepoda; uncultured eukaryote	Copepoda	1.42	0.28	0.08	0.00	0.08
*Candacia armata*	Copepoda	1.29	0.41	0.16	0.01	0.19
OTUs (>1%)		8	9	3	6	3
Total OTUs		296	299	235	261	155

### Comparison against species‐specific qPCR assay

**Figure 6:**
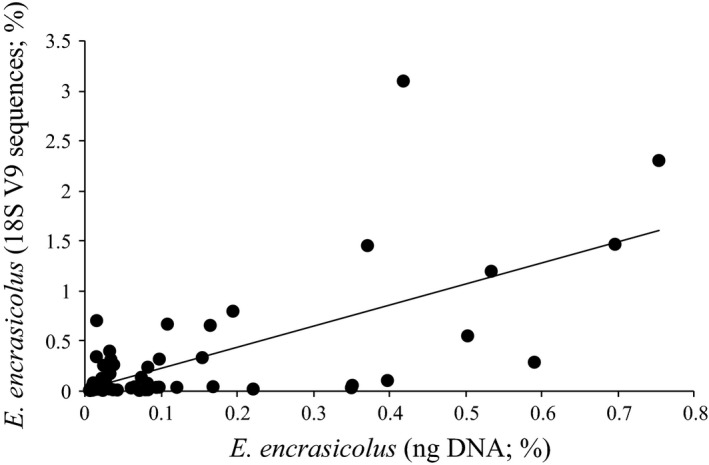
Comparison of metabarcoding‐based detection of European anchovy against species‐specific qPCR assay. The relationship between the amount of European anchovy (*Engraulis encrasicolus*) DNA determined by qPCR and the 18S V9 metabarcoding approach is shown (bottom and left axes, respectively). Linear regression superimposed (*y* = 2.0963*x* + 0.0182; *r* ^2^ = 0.4531).

The presence of DNA of *Engraulis encrasicolus* was detected in all but one of the stomach samples (59/60 positive assays; 98% detection success), including a range of 0.005–0.75% for target DNA amount (Fig. 6). The amount of DNA belonging to E. encrasicolus and estimated in the stomach samples by the qPCR assay and by the metabarcoding approach were significantly correlated (Pearson correlation *r* = 0.67, *P* < 0.01). A similar correlation was observed when the character‐based approach for the clupeid′s OTUs discrimination was used (Pearson correlation *r* = 0.68, *P* < 0.01). Notably, an almost linear relationship was observed when the proportion of *E. encrasicolus* 18S V9 reads was compared among pair of fish stomach sample technical replicates (Pearson correlation *r* = 0.99, *P* < 0.01; Fig. S6).

### Bidirectional (2 × 150) versus single‐direction (1 × 150 bp) 18S V9 amplicon sequencing

On the one hand, bidirectional sequencing increased the number of assigned reads by a factor of 65. The average length of the obtained sequences obtained after the quality filtering step were of 173.8 and 107.1 bp for bidirectional and single‐direction sequencing respectively It has to be noted that the full amplicon was 174 bp length for the tested species (Sanger‐sequenced, Fig. S1 plus primers). On the other hand, the much lower discrepancy against the character‐based assignment approach (Table 4; see Methods for further information) showed the higher assignment reliability of the bidirectional sequencing approach compared to the single‐direction sequencing one.

**Table 4:** Evaluation of the bidirectional sequencing effect on the assignment process′ performance. We retrieved the total number of reads assigned to clupeid′s OTUs when following the described bioinformatics pipeline for both the bidirectional and single‐direction sequencing approaches (except for the merging step in the latter, obviously) as to compare between both set of data and also against a character‐based assignment (see Materials and Methods for further information). The degree of discrepancy (%) between the latter and each of the sequencing approaches is shown for the total number of clupeid reads and also split by each of the clupeid OTUs

Bidirectional sequencing (merged paired‐end reads)				
UCLUST method (close reference assignment; 99 % identity treshold)		Character‐based (3 fixed positions based assignment )		Discrepancy %
OTU	No. reads		No. reads	
*Clupea harengus*	3260	CGC	2290	29.8
*Sprattus sprattus*	964,322	CAT	960,594	0.4
*Sardina pilchardus*	185,591	TGC	182,671	1.6
*Engraulis encrasicolus*	25,830	CGT	23,988	7.1
Total assigned (clupeid species)	1,179,003		1,169,543	0.8
		Others nucleotide combinations (XYZ)	7547	0.6
		Too short for reliable assignment	1913	0.2

Single direction sequencing (forward reads only)				
UCLUST method (close reference assignment; 99% identity treshold)		Character‐based (3 fixed positions based assignment)		Discrepancy %
OTU				
*Clupea harengus*	948	CGC	133	86.0
*Sprattus sprattus*	6943	CAT	3901	43.8
*Sardina pilchardus*	9630	TGC	3763	60.9
*Engraulis encrasicolus*	514	CGT	397	22.8
Total assigned (clupeid species)	18,035		8194	54.6
		Others nucleotide combinations (XYZ)	107	0.6
		Too short for reliable assignment	9734	54.0

## Discussion

Metabarcoding of the 18S V9 region was capable of discriminating the diet of sardine and sprat both in sympatry and, for sardine, along a cross‐shelf transect with contrasting but stable plankton communities (Albaina and Irigoien 2004). Furthermore, the method allowed to distinguish the diel feeding behavior of sardine in the inner shelf region. This is in accord with what is known regarding the capacity of sardine to switch from filtering to particulate feeding modes (e.g. Garrido et al. 2007). For both sardine and sprat, the diet was dominated by medium‐size copepod species although sardines showed a broader prey range (Table 3, Fig. 5). This is also in accord with previously reported microscopy‐based studies (Möllmann et al. 2004; Garrido et al. 2008; Raab et al. 2012; Costalago et al. 2015). Apart from copepods, where visual identification of damaged remains is usually possible, the presence of echinoderms (obviously planktonic larvae), comprising on average 6% of 18S V9 reads within sardine contents, has not been previously noted and illustrates the capacity of metabarcoding to identify the remains of fragile and/or very small organisms which are usually impossible to identify visually in digested stomach contents. Moreover, our results support the idea that sardine may rely on the consumption of protists at times (Garrido et al. 2008). Although targeting a distinct multicopy gene (and organelle), the 18S V9 metabarcoding performance was comparable to the previously tested TaqMan method (qPCR) when quantifying European anchovy DNA remains within fish gut contents (Albaina et al. 2015). This was confirmed by the application of a character‐based approach to discriminate between both predators and the targeted prey (only distinguished by 1–3 variable positions; Fig. S1). This is an important result because it shows that metabarcoding is capable of discriminating OTUs in a reliable way with even a single nucleotide variation within the amplified region.

The power of the 18S V9 metabarcoding approach to estimate relative abundances of a certain taxon at different sample compositions was supported by the significant correlations against the qPCR assay and also when comparing microscopic counts in the artificially assembled (mock) and field plankton samples. As expected, 18S V9 sequences represented a better proxy for biomass distribution than for abundance reflecting the broad range of sizes/stages for plankton taxa potentially encountered in field samples. However, the capacity to reconstruct relative abundances among taxa (within a particular community sample) seemed to be limited to samples dominated by a few taxa i.e. with low evenness (such as MOCK‐D, Fig. 3) (Egge et al. 2013). This is probably a result of the variable 18S rRNA gene copy numbers between different species, up to four orders of magnitude in eukaryotes (Prokopowich et al. 2003). Because of this, metabarcoding multicopy genes (mtDNA, cpDNA, rRNA) is generally considered as a semiquantitative approach (e.g. Amend et al. 2010; Murray et al. 2011) and this was confirmed here. However, the capacity of the 18S V9 metabarcoding approach to describe diel and spatial changes in the relative contribution of prey items in fish diets means that the tool could be of great benefit for improving understanding of fish diets.

### Potential biases affecting the performance of the 18S V9 metabarcoding approach

#### Barcode amplification range

There is typically a trade‐off between barcode amplification efficiency and discriminatory power (e.g. Tang et al. 2012). In this sense, the gold standard of barcoding, the mtDNA cytochrome c oxidase I (MT‐CO1) gene, is severely affected by partial, and a priori undetermined, amplification success (Deagle et al. 2014). On the other hand, a higher amplification range is reported for the 18S rRNA gene, but at a cost of a somewhat reduced taxonomic resolution (Tang et al. 2012; Zhan et al. 2014). However, for diet assessment and, especially, with nonspecialist predators such as filter feeders, it is generally preferable to cover as much of the potential taxonomic diet breadth as possible.

There are alternatives to overcome marker amplification failure including either use of a combination (cocktail) of primers or avoiding the PCR amplification step entirely (metagenomics or metatranscriptomics approaches; Zhou et al. 2013; Srivathsan et al. 2015). However, whilst these technologies develop, the 18S V9 region provides a promising target for metabarcoding due to its reported broad amplification range (e.g. Amaral‐Zettler et al. 2009; de Vargas et al. 2015).

#### Barcode taxonomic resolution

We have shown that, providing there is at least one variable position in the 18S V9, metabarcoding of this region is capable of discriminating species in a reliable way if they are included within the barcode database. In this sense, the taxonomic resolution limit corresponds to the barcode synonymy. However, due to the relatively reduced taxonomic resolution of the 18S V9 region, the availability of full length and high sequencing quality amplicons is crucial. The herein presented method allowed this by totally overlapping the amplicon variable region using PE sequencing and applying a stringent merging step (e.g. Eren et al. 2013). As a result of this, the assignment success (and reliability) was highly improved (Table 4).

#### 18S rDNA copy number variation (CNV)

It is known that the CNV bias associated with multicopy genes limits the quantitative value of the metabarcoding approach (Pompanon et al. 2012). However, for rDNA have been reported correlations between CNV and genome size in eukaryotes (Prokopowich et al. 2003) and, between CNV and cell length and cell biovolume in unicellular organisms (Zhu et al. 2005 and Godhe et al. 2008), which suggests a potential way of addressing this. Another alternative is to use a single‐copy gene as barcode. Although some have already been evaluated for certain taxonomic groups, they consistently showed reduced performance (i.e. lower PCR amplification and sequencing success) compared with multicopy genes (e.g. Schoch et al. 2012 for the largest subunit of RNA polymerase II, second largest subunit of RNA polymerase II, and minichromosome maintenance protein genes in fungi; Pillon et al. 2013 for the *Clerm*2 and *Clerm*4 genes in plants; Stockinger et al. 2014 for the largest subunit of RNA polymerase II gene in fungi). Nevertheless, for the study of degraded DNA, such as is found in stomach contents, the multicopy nature of the barcode is an advantage (King et al. 2008).

#### 18S V9 reference database

A deep (and curated) database is mandatory for the success of any metabarcoding approach and represents another drawback to the use of single‐copy genes where available sequences are scarce. However, regarding 18S V9, SILVA provides a comprehensive and curated database covering the major domains of life (e.g. Hadziavdic et al. 2014; Yilmaz et al. 2014). Adding local species with no previous representation in the database significantly increases the assignment success for locally collected field samples and is recommended when designing a metabarcoding study (e.g. Cowart et al. 2015). Moreover, this allows identifying putative barcode synonymy cases. Regarding the present study, using our custom database the assignment success increased from 65% (SILVA v111 alone) to 76.5% using a 99% identity threshold.

#### 18S V9 sequencing depth

The number of barcode reads retrieved from each sample is critical for the detection of low abundance taxa (e.g. Egge et al. 2013, 2015; Zhan et al. 2013). Besides this, the high sequencing depth achieved in the present study made the use of predator DNA blocking primers unnecessary, if used these can prevent or limit related species amplification (e.g. Piñol et al. 2014). In this sense, the high throughput capacities of the MiSeq platform allowed both the sequencing depth and the number of analyzed samples to be increased compared with other HTS technologies (e.g. Mahé et al. 2015). However, including controls is highly recommended as to evaluate putative cross‐contamination associated to multiplexing (the so‐called mistagging phenomenon; Esling et al. 2015).

#### Other factors

Although common and shared to any potential barcode region and not only the 18S V9 one, other potential biases to be considered in any metabarcoding study would include the DNA extraction bias (the fact that the performance of a certain DNA extraction method can vary with the organism type or even development stage), the inability to discern between dead or alive individuals, in dietary analysis the inability to discriminate secondary predation records, the different bioinformatics′ methods and thresholds for the trimming step, etc.

## Conclusion and Further Work

The 18S V9 broad amplification range, multicopy nature and relatively small size, along with the relatively high assignment success due to both the comprehensive SILVA database (especially when complemented with local key species) and the bidirectional sequencing approach, makes the herein described method suitable for the analysis of degraded DNA.

This is supported here by demonstration of its capacity to discriminate the diet of two sympatric zooplanktivorous fish species targeting a similar prey spectrum. Furthermore, it distinguished both spatial and temporal (diel) shifts in relative proportions of different taxa in the diet. By comparing metabarcoding results against other proven techniques such as microscopy and qPCR, present study′s results suggest that 18S V9 metabarcoding is at least as sensitive but with much higher throughput capacity.

Further developments should explore the combination of 18S V9 with higher taxonomic resolution barcode/s. In this sense the 18S V1‐V2 region (Fonseca et al. 2010; Lindeque et al. 2013; Zimmermann et al. 2014; Brown et al. 2015) is a promising candidate to combine with the 18S V9 one due to a ~400–500 bp amplicon length making it possible to better discriminate between species (Dunthorn et al. 2012 and Fig. S7 for herein included copepod and euphausiid species) without including a distinct barcode CNV bias. Besides this, the availability of contrasting length barcodes could allow to infer the degree of DNA damage (i.e. digestion time; Deagle et al. 2006), a key aspect in the forensics field.

## Data Accessibility

GenBank accession numbers for the herein produced 18S V9 Sanger sequences: KP768123‐KP768151 (see also Table S2).

Metabarcoding data (MiSeq′s 18S V9 quality filtered merged reads; .fasta file) and mapping file (with library tags and sample correspondence; .xls file) available from the Dryad Digital Repository: http://dx.doi.org/10.5061/dryad.2p1t8.

## Conflict of Interest

None declared.

## Supporting information


**Table S1.** Fish hauls. Data on fish haul including date, local time, depth (both haul and bottom) are shown along with the number and length of analysed fish.

**Table S2**. GenBank accession numbers, including origin details, for every taxon used for *in silico* testing of 18S rRNA V9 taxonomic resolution and, to create the local reference database.

**Table S3**. Synonymy between local and SILVA database. The herein generated sequences from local species (Sanger) were compared against SILVA database with BLASTN algorithm. Those hits covering at least 64 nucleotides of the 18S V9 region and with a 100% identity are shown. GenBank accession number and 18S V9 covered length (bp) are shown within brackets.

**Figure S1**. Clupeid fish 18S V9 region.
**Figure S2.** 18S V9 maximum likelihood (ML) tree.
**Figure S3**. Field samples.
**Figure S4**. Multivariate analysis of sardine and sprat diets using the open reference method for OTU assignment.
**Figure S5.** Multivariate analysis of fish technical replicates.
**Figure S6.** Metabarcoding‐based detection of *Engraulis encrasicolus* in stomachs′ technical replicates.
**Figure S7** 18S V1‐V2 maximum likelihood (ML) tree.
