# Supplementary material for: 18S rRNA V9 metabarcoding for diet characterization: a critical evaluation with two sympatric zooplanktivorous fish species
Source: Ecol Evol. 2016 Feb 19;6(6):1809–24. doi: 10.1002/ece3.1986 (PMC4801955; doi:10.1002/ece3.1986)

**Supporting Information for online publication**

**-Supplementary tables´ legends.**

**Table S1.** Fish hauls. Data on fish haul including date, local time, depth (both haul and bottom) are shown along with the number and length of analysed fish.

**Table S2**. GenBank accession numbers, including origin details, for every taxon used for *in silico* testing of 18S rRNA V9 taxonomic resolution and, to create the local reference database.

**Table S3**. Synonymy between local and SILVA database. The herein generated sequences from local species (Sanger) were compared against SILVA database with BLASTN algorithm. Those hits covering at least 64 nucleotides of the 18S V9 region and with a 100 % identity are shown. GenBank accession number and 18S V9 covered length (bp) are shown within brackets.

**-Supplementary figures and legends.**

**Figure S1**. Clupeid fish 18S V9 region. Alignment showing the location of variable positions between *Engraulis encrasicolus*, *Sardina pilchardus*, *Sprattus sprattus* and *Clupea harengus* 18S V9 region (excluding primers; 16 and 24 bp for Forward and Reverse one, respectively). Two individuals were sequenced for each species.


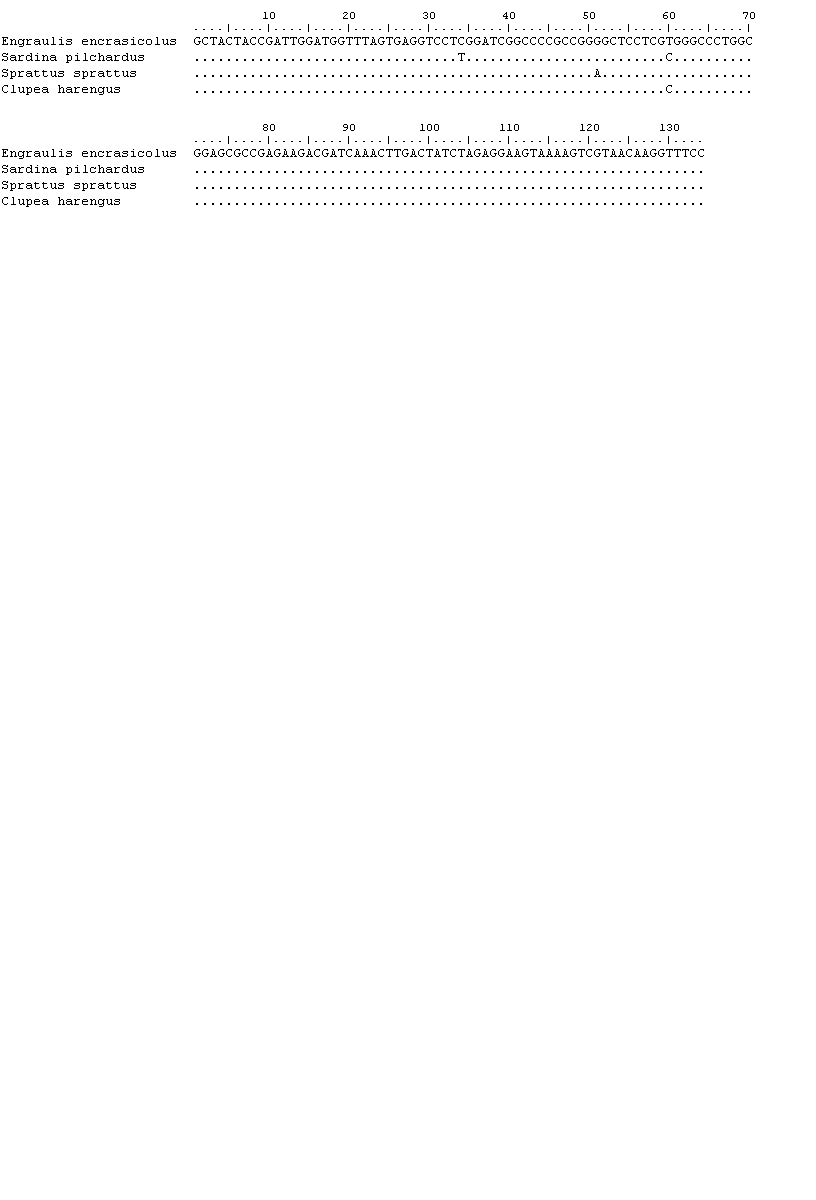


**Figure S2.** 18S V9 maximum likelihood (ML) tree. ML tree constructed with the best-fit model of evolution (K2+G) and 500 bootstrap replicates. Seventy seven 18S V9 sequences from thirty five different species were included in the analysis (see Methods for further details). A 146 bp alignment (MUSCLE) was used. The scale bar indicates substitutions per nucleotide position.


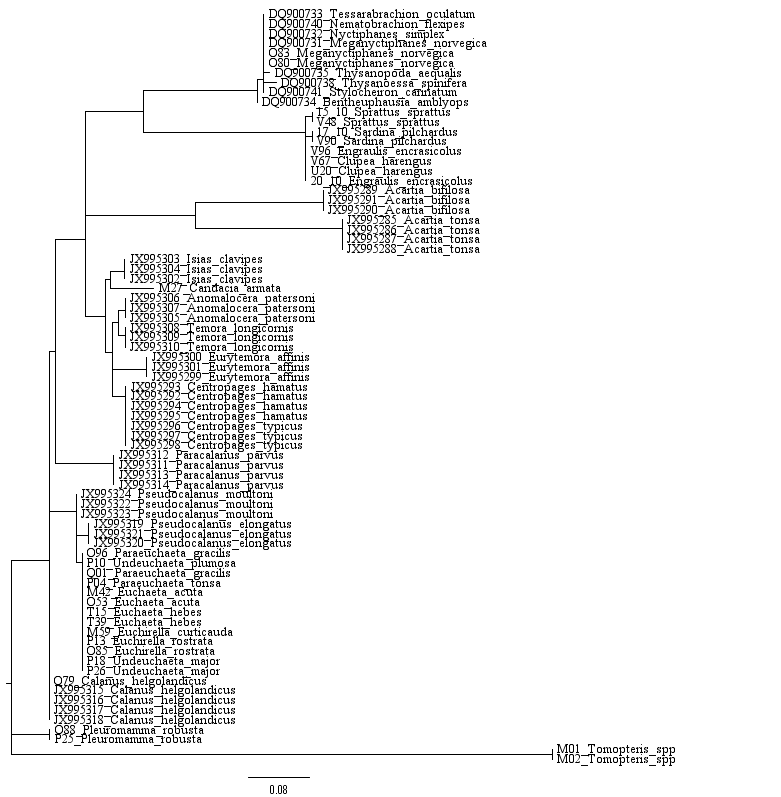


**Figure S3**. Field samples. Relative abundance of a) microscopy counts, b) estimated biomass (C dry weight) and, c) 18S V9 obtained reads, for selected taxonomic categories within field samples. Five technical replicates were sequenced (1-5, bottom graph). No bias in OTUs distribution was reported for the technical replicates (Kruskal-Wallis test). Legend superimposed.

**Figure S4**. Multivariate analysis of sardine and sprat diets using the open reference method for OTU assignment. Detrended correspondence analysis (DCA) generated considering OTUs comprising ≥ 0.5 % of 18S V9 reads within any of the analyzed stomachs (a total of 91 OTUs). The five fish hauls (n= 84 stomachs) are represented with distinct symbols. Empty squares corresponded to shelf-break collected sardines (haul A), rhombus did to outer shelf ones (haul B) and down- and up-oriented triangles represented, respectively, haul C and D sardines (both in the inner shelf area). Finally, the sole sprat haul (inner shelf, haul E) was represented by black dots. Fishing was performed during daylight except for haul D which took place at midnight.

**Figure S5.** Multivariate analysis of fish technical replicates. Two independent libraries were generated for 32 sardines´ stomachs (n = 64). The detrended correspondence analysis (DCA), considering OTUs comprising ≥ 0.5 % of 18S V9 reads within any of the analyzed stomachs, is shown. Fish hauls symbols as in Figure 4. Full and empty symbols with correlative numbering (from 2 to 65) represent replicate pairs. The three more distant replicate pairs in the plot (44 -4 5, 48 - 49 and 56 - 57) are depicted with arrows as to illustrate this.

**Figure S6.** Metabarcoding-based detection of *Engraulis encrasicolus* in stomachs´ technical replicates. The relationship between the amounts of European anchovy DNA determined by the 18S V9 metabarcoding approach in each pair of stomachs´ technical replicates is shown. Linear regression superimposed (y = 0.84x + 0.0447; r² = 0.9833).

**Figure S7** 18S V1-V2 maximum likelihood (ML) tree. ML tree constructed with the best-fit model of evolution (T92+G) and 500 bootstrap replicates. Fifty four 18S V1-V2 sequences from twenty four different species (those of Laakmann *et al.* 2013 and GenBank´s popset 117414780) were included in the analysis. A 390 bp alignment (MUSCLE) was used. The scale bar indicates substitutions per nucleotide position. 18S V1-V2 region as delimited by Fonseca *et al.* (2010) primers.


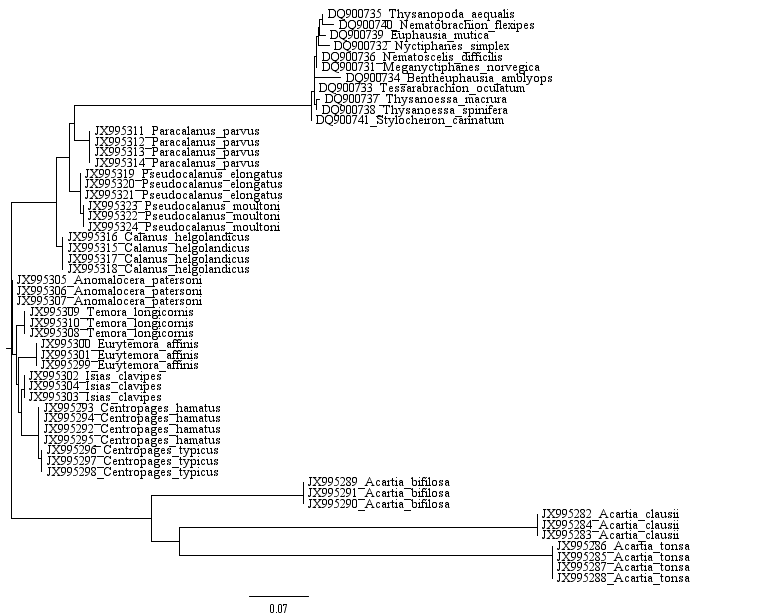

Supplement: Supplementary file 4 — Figure S1. Clupeid fish 18S V9 region. Figure S2. 18S V9 maximum likelihood (ML) tree. Figure S3. Field samples. Figure S4. Multivariate analysis of sardine and sprat diets using the open reference method for OTU assignment. Figure S5. Multivariate analysis of fish technical replicates. Figure S6. Metabarcoding‐based detection of Engraulis encrasicolus in stomachs′ technical replicates. Figure S7 18S V1‐V2 maximum likelihood (ML) tree. [file ECE3-6-1809-s004.docx]
